# Understanding the influence of patient demographics on disease severity, treatment strategy, and survival outcomes in merkel cell carcinoma: a surveillance, epidemiology, and end-results study

**DOI:** 10.18632/oncoscience.358

**Published:** 2017-09-14

**Authors:** Harib H. Ezaldein, Alessandra Ventura, Nicolaas P. DeRuyter, Emily S. Yin, Alessandro Giunta

**Affiliations:** ^1^ University of Washington, Department of Surgery, Seattle, Washington, USA; ^2^ University of Rome Tor Vergata, Department of Dermatology, Rome, Italy; ^3^ Yale University School of Medicine, Department of Dermatology, New Haven, Connecticut, USA

**Keywords:** merkel cell carcinoma, outcomes, socioeconomic, SEER database, nationwide study

## Abstract

**Objective:**

To identify trends in patient presentation and outcomes data that may guide the development of clinical algorithms on Merkel Cell Carcinoma (MCC).

**Methods:**

We performed a retrospective cohort study searching in the National Cancer Institute's SEER registry for documented MCC cases from 1986-2013. No exclusion criteria were applied. We hereby identified 7,831 original MCC entries. Demographics, staging, and socioeconomic characteristics were identified and treatment modality likelihoods and survival data were calculated via logistic regression and Kaplan-Meier statistical modeling.

**Results:**

Concerning tumor localization, 44.5% (n= 3,485) were located on the head and neck, and 47.8% were located on the trunk and extremities (n= 3,742). Male and younger patients are more likely to receive radiation than surgery with no differences seen among patient race. Caucasians and “Other” races both showed higher overall survival than African American patients. States with higher median household income levels demonstrated survival advantage. Income quartiles yielded no differences in surgical or radiotherapy interventions. Moreover, patients who forego radiotherapy had a poorer overall survival.

**Limitations:**

Generalizability of SEER data, potential intrinsic coding inconsistencies, and limited information on patient comorbidities, sentinel lymph node and surgical margin status are major limitations. There is no information regarding medical intervention such as systemic chemotherapy or immunotherapy. Recoding efforts are inconclusive regarding variables such as tumor infiltrating lymphocytes, mutations, or immunosuppression status, which are well-documented for other cancers within the database.

**Conclusion:**

MCC lesions of the head and neck region, lower income quartiles, and African American race are associated with higher mortality. MCC patients have a median household income that is significantly higher than national values with no significant difference in subsequent treatment modalities (surgery or radiotherapy) based on socioeconomic markers. A lack of radiotherapy is associated with higher mortality.

## INTRODUCTION

Merkel cell carcinoma (MCC) is a rare and aggressive neuroendocrine malignancy often associated a poor prognosis that was described by Toker in 1972 as a trabecular carcinoma of the skin [1, 2, 3, 4]. It has been proposed that the incidence of MCC is increasing, with nearly 1,600 new cases per year in the United States, an observation perhaps related to improvements in diagnostic strategy and skin cancer awareness [5, 6, 7, 8, 9, 10]. MCC incidence is also 5- to 13-fold greater in immunosuppressed populations including HIV and solid organ transplant patients [6, 7, 8, 9].

MCC clinically presents as a painless, raised, reddish-blue nodule that rapidly progresses and frequently affects Caucasian patients over 50 years of age at sun-exposed sites, such as the head and neck. African Americans patients comprise less than 1% of the affected population with a preponderance of tumors located at the extremities [6, 11, 12, 13]. These classic clinical features can be summarized with the useful acronym AEIOU (asymptomatic, expanding rapidly, immunosuppression, older than 50 years of age, UV exposure on fair skin) [14]. The diagnosis of MCC can be challenging, with a clinical differential that includes nodular basal cell carcinoma or amelanotic melanoma, with up to 30% of MCCs are misdiagnosed as metastatic oat-cell carcinoma, particularly at tumor onset [3, 6].

Though the precise pathophysiological mechanisms leading to tumor onset, progression, and occasionally spontaneous regression, are still debated, a significant association between MCC and ultraviolet radiation exposure and Merkel cell polyomavirus (MCPyV) infection has been observed. In particular, a chromosomal localization of MCPyV, specifically ST and LT viral sequences, which behave as oncoproteins, is significantly expressed in all MCCs [3]. Ultraviolet radiation may play a role in the development of MCC due to an immunosuppressive effect related to the increase of modulating cytokines such as interleukin-10 and tumor necrosis factor alpha [15].

Multimodal treatment strategies exist for MCC, including wide local excision with or without sentinel lymph node biopsy, Mohs micrographic surgery, radiotherapy, chemotherapy, and anti-PD-1 immunotherapy in selected cases [16]. Despite aggressive therapeutic approaches, MCC has a high local recurrence and mortality rate, with a median progression-free survival of approximately three months [1, 4]. However, approximately half of MCC tumors express PD-L1 on tumor cells and PD-1 on T-lymphocytes, rendering them highly susceptible to PD-1 inhibitors; PD-1 expression is also correlated with enhanced survival [2, 5].

Strong associations have been shown between socioeconomic status (SES) and survival in patients with skin cancer [11, 14]. Given the increasing incidence of MCC, it is imperative to expand our understanding of prognostic factors and other disease features that may influence survival.

Herein, we characterize the effects of clinical and socioeconomic markers on disease management, progression, and survival for all diagnosed MCC cases between 1986-2013 using data from the National Surveillance, Epidemiology, and End Results (SEER) program database. Our objective was to identify outcome tendencies that may guide the development of future clinical algorithms as newer clinical trial data and treatment options become available for MCC.

## RESULTS

Approximately 7,831 MCC cases were retrieved from the SEER Database (Table 1). The patient population was negatively skewed and largely above the age of 60 years, with a median age of 77 years, and majority being male (62%). Median income of this dataset ($59,710) is significantly higher than the national median household income for 2015 ($56,516) [17]. Of the characterized lesions, 44.5% (n= 3,485) were located on the head and neck, and 47.8% were located on the trunk and extremities (n= 3,742). The majority of tumors were not characterized by AJCC staging (77.65%, n=6,081). While 39.91% (n=3,125) of lesions in our dataset had a maximal dimension tumor size measuring less than 2 cm, the majority (56.93%) was not characterized with regard to sizing (n=4,458). Lymph node biopsies or excisions were utilized in 30.7% of studied lesions. Beam radiation was used for 43.9 % (n= 3,434) of lesions, while no radiation was received for 53.2% (n= 4,168) of cases.

**Table 1:** Summary of Patient-Level Demographic and Disease-specific Data

Age (years)	N (=7,831)	% of Total
<60	869	11.10%
≥60	6,962	88.90%
Mean	75	
Median	77	
Std. Dev	11.8	
Range	11-105	
IQR, 25%-75%	68-84	
**Race**		
Black	88	1.10%
Other (American Indian/AK Native, Asian/Pacific Islander)	207	2.60%
Unknown	99	1.30%
White	7,437	95.00%
**Sex**		
Male	4,855	62.00%
Female	2,976	38.00%
**Income**		
Mean	$61,700	
Median	$59,710	
Std. Dev	$14,754	
IQR, 25%-75%	$53,250-$73,040	
Range	$20,000-$106,520	
**Radiation**		
Beam radiation	3,434	43.90%
Combination of beam with implants or isotopes	5	0.10%
None or Refused	4,168	53.20%
Radiation, method or source not specified	55	0.70%
Radioisotopes/Radioactive implants	6	0.10%
Unknown	163	2.10%
**Primary Site**		
Head and Neck	3,485	44.50%
Trunk and Extremities	3,742	47.80%
Unspecified	604	7.70%
**Stage**		
I	678	8.66%
II	311	3.97%
III	568	7.25%
IV	193	2.46%
Unknown	6,081	77.65%
**Tumor Size**		
<2 cm	3,124	39.91%
2-5 cm	37	0.47%
>5 cm	8	0.10%
No tumor found	203	2.59%
Unknown	4,458	56.93%
**Regional LN Scope Categories**		
Regional LN Bx only	114	1.5
At least 1 regional LN removed	1007	12.9
Sentinel LN bx only	1081	13.8
Sentinel LN and regional removed	199	2.5
None	2749	35.1
Unknown	2681	34.2

The effects of demographic and disease presentation factors on the odds of receiving surgery or radiation was also analyzed (Table 2). Male and young patients were more likely to receive radiation than surgery with no appreciable difference seen with regard to patient race. There was also no observed effect of income level on subsequent treatment modalities. Lesions of the trunk or with a tumor size ≤2 cm were associated with higher rates of surgical intervention than radiation when compared to lesions of the head and neck. Intermediate lesions (2-5 cm), Stage III, and Stage IV lesions were more likely to receive radiation over surgery (Table 2).

**Table 2:** Patient Demographic, Clinical Presentation, and Likelihood of Undergoing Surgery or Radiotherapy

	Odds Ratios		
		*Radiation*	*Surgery*
**Sex**			
	*Male*	1.341	0.921
**Age**			
	*60+*	0.511	0.99
**Race**			
	*Black*	0.953	0.37
	*Other*	1.649	0.586
	*Unknown*	0.445	0.168
**Income**			
	*Q2*	0.754	0.891
	*Q3*	1.045	0.646
	*Q4*	0.906	0.898
**Primary Location**			
	*Trunk and Extremities*	0.955	1.513
	*NOS*	0.83	0.086
**Size**			
	*2-5cm*	3.451	0.586
	*>5cm*	0.156	0.052
	*Primary Tumor NOS*	1.301	0.039
	*Unknown*	0.771	0.264
**Stage**			
	*Stage 2*	1.594	0.408
	*Stage 3*	3.541	0.414
	*Stage 4*	1.368	0.107

Significant values are in red.

**Figure 1:**
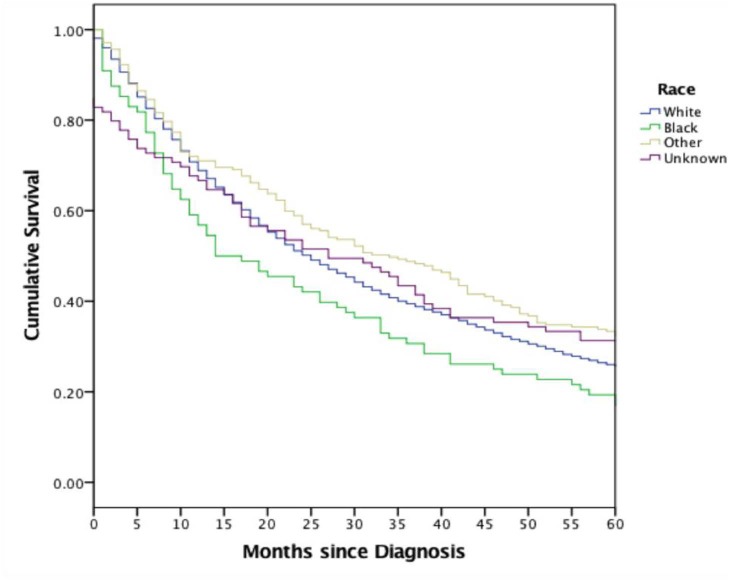
Survival analysis among races studied including white, black, other, and unknown White patients show higher survival than Blacks, with patients under the “Other” category demonstrating the highest overall survival.

**Figure 2:**
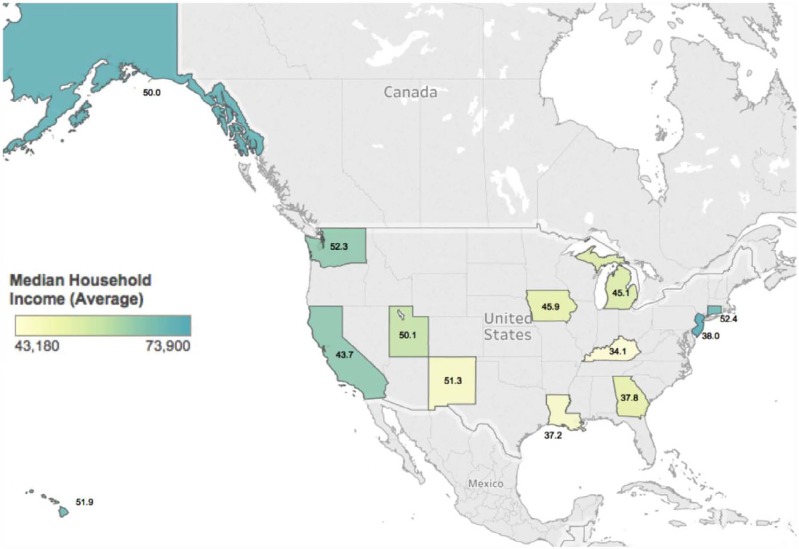
A geographical portrayal of median survival in months in SEER-participating states States are colored on a spectrum based on reported median household income, with each state numbered according to median survival months.

Patient demographic and tumor characteristics were also significantly associated with one-, three-, and five-year survival (Table 3). White patients had higher survival rates over a five-year period after diagnosis than Blacks, while patients whose race was categorized as “Other” enjoyed the highest survival rate overall (Figure 1). Patient who lived in states with higher median incomes had higher median survival times (Figure 2). Older patients had increased mortality at all time periods, an effect that increased at the five-year mark. Higher quartiles of income (Q3 and Q4) had a delayed protective effect, seen at the three- and five-year time points. Smaller lesions (<2 cm) and those located on the trunk and extremities were associated with higher survival rates. Lesions receiving combination radiotherapy were associated with significantly increased mortality in the first year of diagnosis, while patients who did not receive radiation had higher mortality across all time periods.

**Table 3:** Mortality Odds (1-, 3-, and 5-year) Data in Relation to Patient-level Demographic, Tumor staging, and Treatment Data

Mortality Odds-Ratios				
		*1 year*	*3 year*	*5 year*
**Sex**				
	*Male*	1.202	1.396	1.471
**Age**				
	*60+*	1.915	1.908	2.437
**Race**				
	*Black*	1.803	1.578	1.798
	*Other*	0.957	0.743	0.792
	*Unknown*	1.156	0.918	0.848
**Income**				
	*Q2*	0.949	0.931	0.912
	*Q3*	0.91	0.831	0.795
	*Q4*	0.909	0.848	0.83
**Primary Location**				
	*Trunk*	0.884	0.795	0.8
	*NOS*	1.683	1.483	1.559
**Size**				
	*2-5 cm*	1.421	0.991	2.34
	*>5cm*	3.078	3.33	3.34
	*Primary Tumor NOS*	0.658	1.01	0.787
	*Unknown*	0.641	0.505	0.382
**Radiation**				
	*Combination*	4.986	1.415	2.377
	*None*	1.365	1.372	1.361
	*NOS*	0.995	0.932	0.837
	*Isotope/Implant only*	1.432	1.697	0.95
	*Unknown*	1.349	1.096	1.161

Significant values in red.

## MATERIALS AND METHODS

Data was obtained from the National Cancer Institute's SEER registry for diagnosis of MCC from 1986-2013 (November 2015 update), accessed on June 15th, 2016. Patient demographics, tumor characteristics, and treatment options were analyzed. Demographics include age at diagnosis, gender, race, county and state of diagnosis, survival in months, and median income. Tumor characteristics include size and location of primary tumor, lymph node involvement, and American Joint Committee on Cancer (AJCC) staging. Treatment strategies are divided into surgical intervention and radiation therapy, with several subcategories for the latter.

No exclusion criteria were applied to our dataset. Age and median income were analyzed as both categorical and continuous variables. Survival in months was analyzed as a continuous variable. Gender, race, county and state of diagnosis, tumor characteristics, and treatments were categorical variables.

A Kaplan-Meier curve was created to assess one, three, and five-year survival risks. Logistic regression was used to estimate mortality odds ratios within time intervals for given patient demographics, tumor characteristics, and radiation treatments. Logistic regression was used to also estimate surgery versus radiation treatment given specific patient demographics and tumor characteristics. When determining time-point survival odds ratios, the following variables were standardized: age compared to <60 years, race categories compared to “White”, primary tumor location data was standardized to head and neck, tumor size was compared to “<2 cm”, types of radiation were compared to “beam radiation” category, and all stages were standardized to Stage 1 lesions. A map was created to illustrate the geographical and median household state income association with MCC median survival lengths (in months). Statistical analysis was performed using SPSS Version 24 (IBM, New York, USA) and Tableau Version 9 (Tableau Software, Washington, USA). All values with a two-tailed p-value less than 0.05 were considered significant.

## CONCLUSIONS

In this retrospective SEER study, we report on one of the largest epidemiology registries of MCC to describe the effects of clinical and socioeconomic factors on disease management, progression, and survival for all diagnosed MCC cases between 1986 and 2013. We showed that lesions of the head and neck region, lower income quartiles, and African American race are associated with higher short-term and long-term mortality. Although our data indicates that lower median income predicts shorter survival, correlating with other types of skin cancer [18, 19, 20, 21], modes of subsequent treatment were not significantly different with univariate analysis of race or income level. This intriguing finding could be confounded by the overall predilection of MCC towards Caucasian patients, potentially resulting in inadequate sample size effects for lower socioeconomic patients. Despite advances in diagnosis and treatment strategies, such as immunotherapeutic targeting [3, 7], a significant number of cases in our study originate from unknown primaries, correlating with literature cases [22, 23].

Surprisingly, we note that income quartiles within our cohort are significantly above nationwide median income, which may be in accordance with previous studies showing an increasing rate of developing skin cancer with higher incomes or educational level [20, 24, 25, 26]. Some have attributed social trends such as perceptions of tanned skin and increased outdoor activity as historical correlations [24]. Hausauer and colleagues showed higher relative and absolute risks of developing melanoma with increasing socioeconomic status quintiles over time in all groups under study [27]. It was also observed in that study that women living in more affluent neighborhoods were diagnosed with skin cancer 70% more often than their counterparts in lower income residences. Even with this increased risk of skin cancer development, there is an observed protective effect with regard to overall survival as seen in our study and others [20, 21, 26].

Head and neck lesions are also shown to predict poorer overall survival, perhaps due to increased histopathological aggressiveness or regional lymphovascular network [28, 29, 30, 31]. Inadequacy of margins, complicated facial anatomy, and a questionable value of sentinel lymph node status are also implicated [32, 33]. Fields and colleagues showed lymph node involvement in 29% of their cohort, for which sentinel lymph node biopsy was unrelated to recurrence or survival [32]. It has also been shown that sentinel lymph node positivity has been associated with better overall and disease-free survival when combined with adjuvant radiotherapy [34].

Several key findings help improve our notion of characterizing overall risk, though our study is limited by generalizability of database research. SEER data is representative of ~10% of the United States population, limiting geographical trend assessments, though its socioeconomic measures are representative of the overall population [35, 36]. Additionally, the SEER data collection centers can inherently present a data selection bias, due to the higher volume or level-of-care attributes seen at the tertiary- or quaternary-centers. This can result in larger amounts of patients with late clinical presentations, such as nodal involvement, or more aggressive disease pathologies. The coding of events is not primarily intended for sophisticated research perspectives, which may lead to a sub-optimal investigation of variable effects such as tumor staging, pathology findings, expressed tumor markers, surgical excision margins, or medical treatment modalities [36, 37]. Patient comorbidities and surgical pathology information (margins, sentinel lymph node status) are also absent from the dataset. Such variables, especially in a cancer that affects older patients, can guide the treatment strategies for unique cases that may not be covered by the database. Nearly 80% of the MCC entries do not have known AJCC stages, possibly due to staging revisions over the years, though this deficiency can be partially leveraged with more modern staging criteria based on tumor size and lymph node involvement. There is also no subcategorization for medical therapies such as systemic chemotherapy or immunotherapy. Additionally, follow-up time is curtailed in recently diagnosed patients (2011+). Several recoding efforts also provide inconclusive data regarding site-specific variables such as tumor infiltrating lymphocytes or immunosuppression status, variables that are characterizable for other cancers within the same database. Future iterations will need to include a more modernized level of data such as mutational data, PD-1 status, and response to various aforementioned therapies.

Our findings demonstrate that MCC is a rare condition, predominantly in Caucasian patients, with a worse prognosis when associated with head-and-neck lesions, an age over 60, or patients in lower income quartiles. Thus, a correlation exists between clinical presentation, socioeconomic level and overall survival, with no observable means relating to treatment strategy, that may point to earlier diagnosis and enhanced awareness as implicated causes [21]. Our study significantly expands on factors that influence MCC treatment and survival, extending into the most updated data from the national SEER registry. A number of factors remain understudied, including nodal status, immunosuppression, and coordination of surgery with chemoradiation, but our findings contribute to the understanding of effective diagnostic and survival prediction factors that can be incorporated into future MCC staging algorithms.
